# Effect of tidal volume and positive end-expiratory pressure on lung hysteresis of healthy piglets

**DOI:** 10.1186/cc13476

**Published:** 2014-03-17

**Authors:** DT Andreis, M Milesi, P Pugni, F Nicosia, GE lapichino, M Monti, B Comini, E Votta, A Protti, L Gattinoni

**Affiliations:** 1 Universitá degli Studi di Milano, Milan, Italy; 2 Fondazione IRCCS Cá Granda - Ospedale Maggiore Policlinico, Milan, Italy; 3 Politecnico di Milano, Milan, Italy

## Introduction

Growing evidence suggests that, as long as the total lung capacity is not overcome, dynamic (that is, tidal volume, VT) is more injurious than static (that is, positive end-expiratory pressure, PEEP) lung deformation [1]. Because the lung behaves like a viscoelastic body [2], hysteresis may play a role in the development of ventilator- induced lung injury. The aim of the study was to investigate the effects of increasing VT or PEEP on lung hysteresis.

## Methods

In eight healthy piglets we measured total hysteresis and the peak inspiratory pressure (Ppeak) while randomly increasing VT (with no PEEP) or PEEP (with fixed VT). P1 was extrapolated from the drop in airway pressure during an end-inspiratory pause [3]. Hysteresis attributable to lung parenchyma was computed as: total hysteresis - ((Ppeak - P1) x VT).

## Results

**Figure 1:**
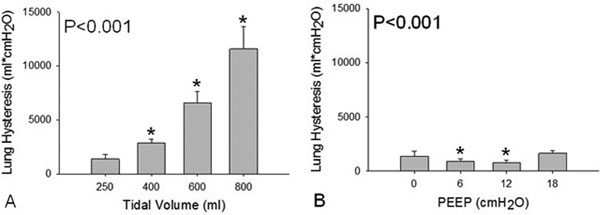
**Lung hysteresis as a function of VT (A) and PEEP (B)**. *P < 0.05 versus VT 250 ml or PEEP 0 cmH_2_O (Holm-Sidak method).

The main findings are shown in Figure 1. *P *values refer to oneway repeated-measures analysis of variance.

## Conclusion

Lung hysteresis increases with VT, but not with PEEP Further studies are needed to prospectively evaluate the role of lung hysteresis in the pathogenesis of ventilator-induced lung injury.
